# Development of a Diagnosis and Evaluation System for Hemiplegic Patients Post-Stroke Based on Motion Recognition Tracking and Analysis of Wrist Joint Kinematics

**DOI:** 10.3390/s20164548

**Published:** 2020-08-13

**Authors:** Subok Kim, Seoho Park, Onseok Lee

**Affiliations:** 1 Department of Computer Science & Engineering, Graduate School, Soonchunhyang University, 22 Soonchunhyang-ro, Asan 31538, Korea; tnqhr93@sch.ac.kr; 2 Department of Medical IT Engineering, College of Medical Sciences, Soonchunhyang University, 22 Soonchunhyang-ro, Asan 31538, Korea; psh8358@naver.com

**Keywords:** leap motion controller, box and block test, hand tracking

## Abstract

An inexperienced therapist lacks the analysis of a patient’s movement. In addition, the patient does not receive objective feedback from the therapist due to the visual subjective judgment. The aim is to provide a guide for in-depth rehabilitation therapy in virtual space by continuously tracking the user’s wrist joint during Leap Motion Controller (LMC) activities and present the basic data to confirm steady therapy results in real-time. The conventional Box and Block Test (BBT) is commonly used in upper extremity rehabilitation therapy. It was modeled in proportion to the actual size and Auto Desk Inventor was used to perform the 3D modeling work. The created 3D object was then implemented in C # through Unity5.6.2p4 based on LMC. After obtaining a wrist joint motion value, the motion was analyzed by 3D graph. Healthy subjects (23 males and 25 females, n = 48) were enrolled in this study. There was no statistically significant counting difference between conventional BBT and system BBT. This indicates the possibility of effective diagnosis and evaluation of hemiplegic patients post-stroke. We can keep track of wrist joints, check real-time continuous feedback in the implemented virtual space, and provide the basic data for an LMC-based quantitative rehabilitation therapy guide.

## 1. Introduction

In recent years, stroke, traumatic brain injury, dementia, and so on have been increasing due to increased life expectancy [1,2,3]. Approximately 85% of patients with stroke experience hemiplegia which is one of the biggest causes of poor quality of life as it interferes with the normal use of other parts of the body due to motor damage in the upper extremities [4,5]. In addition, upper extremities are more difficult to recover than lower extremities. Thus, the therapist has the primary goal of making possible the recovery of the affected upper extremities [5,6,7]. Task-oriented training is a very beneficial therapy for upper extremity rehabilitation [8,9,10]. Intensive repeated training is also an essential therapy for nerve rehabilitation interventions as it can help nerve regeneration and upper extremity function enhancement [9,11,12,13].

However, conventional rehabilitation therapy is tedious and environmentally limited. In addition, it requires experience and skills from the therapist [14,15,16]. To solve this problem, virtual reality (VR) training is available as it enables real-time three-dimensional (3D) observation by moving part of the user’s body in virtual space. It also enables subjects to perform high intensity, repetitive, and task orientated training [17,18,19,20]. However, it is difficult to track hand position motion for the upper limbs during VR-based training, although hand position motion is important for function recovery in hemiplegic patients [21,22]. In addition, VR training has limitations in that it can cause dizziness due to difficult tasks [23].

The Leap Motion Controller (LMC; Leap Motion, Inc., San Francisco, CA, USA), a new device for rehabilitation therapy, can avoid limitations of existing methods [24]. Using LMC, it is possible to capture and track finger movements. Recently, classification techniques have been used for applications using LMC according to hand recognition [25,26]. In particular, it is possible to analyze sign language through qualitative pose measurement based on joints tracked in hand gestures [25]. In addition, LMC is being introduced as an assisted device capable of providing a rehabilitation therapy platform by real-time monitoring of patient recovery [26]. It is possible to analyze unique movement patterns that are based on rehabilitation by automatically extracting features of patient hand movements [26]. Based on this, the LMC can be extended to rehabilitation by analyzing the movement of a sophisticated wrist joint in immersive reality. It is also possible to perform appropriate neuro-rehabilitation therapy in task-oriented of upper extremity even in a virtual environment [27,28]. In addition, the range of motion (ROM) measurement of the hand through LMC allows the evaluation of muscle tone in terms of rehabilitation therapy [29]. According to previous studies, LMC-based rehabilitation training is an optimal rehabilitation therapy based on neuroplastic theory. It has been proven to be a more effective therapeutic approach than conventional rehabilitation therapy [5].

However, LMC rehabilitation training is still focused as a therapeutic approach. In addition, an inexperienced therapist cannot provide objective feedback to the patient by analyzing only the visual judgment of the patient’s movement. Thus, neither the therapist nor the patient receives proper feedback. The therapist needs various techniques and skills to identify patients, motivate them to participate in therapy programs, use appropriate rehabilitation techniques, and make system developments available to perform effective rehabilitation therapy. In the objective 3D analysis of the wrist joint using LMC, it is important to secure medical image data in the subjective rehabilitation field. Therefore, the objective of this study was to provide a guide for in-depth rehabilitation therapy in virtual space by continuously tracking a user’s wrist joint during LMC activities and resent basic data to confirm steady therapy results in real-time.

## 2. Materials and Methods

### 2.1. Participant

Healthy adults (23 males and 25 females, n = 48) in their twenties (22.71 ± 1.65 years) who had no upper extremity musculoskeletal disease during the last three months were included in this study. Experiments were conducted on all participants after receiving experimental methods and prior informed consent.

### 2.2. Box and Block Test (BBT)

**Figure 1:**
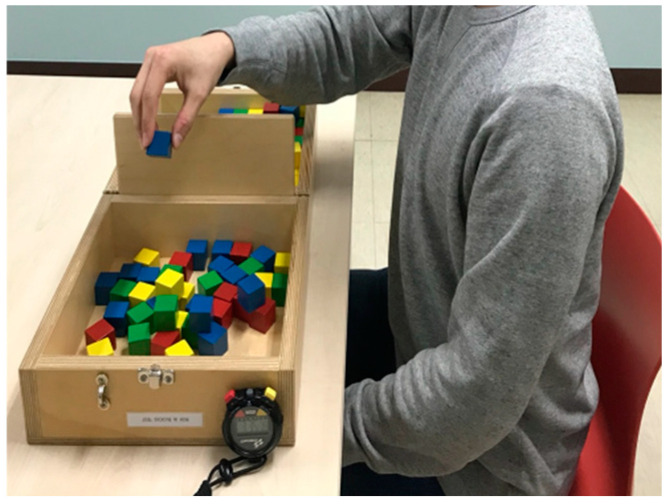
Conventional BBT. Analysis of hand motion of post-stroke hemiplegia patients.

The Box and Block Test (BBT) was developed by A. Jean Ayres, and Patricia Holser Buehler uses blocks to assess the hand motor ability of adult cerebral palsy [30]. It is simple for people who lack hand motor ability to use. BBT was then modified by Patricia Holser Buehler and Elizabeth Fuchs into boxes. For the BBT, the subject is asked to move 150 wooden blocks in one box to another box across a partition from a box with a partition between two boxes (Figure 1). The number of blocks moved is then counted. It is the most important test to judge the hand motor ability of patients with a nervous system injury by comparing the transferred number with a previously established evaluation index [31].

### 2.3. Study Designs and Development Environments

Augmented reality (AR) is a technique that has advantages of sensory engagement, interaction, and autonomy [32]. AR can induce special senses such as tactile sense, auditory sense, smell sense, taste sense, and somatosensory of the user into a spatial system close to reality [33]. In addition, it can provide immediate feedback to the user who has experienced the virtual environment. It can operate autonomously according to the user’s movement.

**Figure 2:**
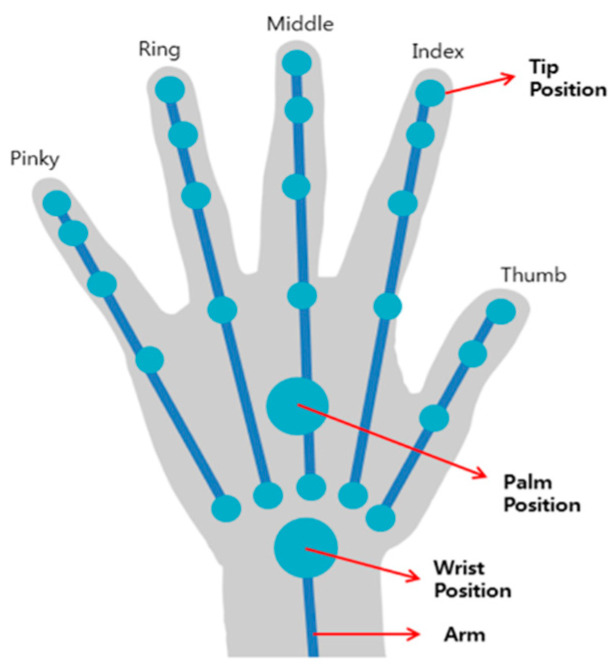
LMC wrist joint recognition range. LMC recognizes the upper extremity in real-time and makes it possible to know the movement pattern of the user.

Rehabilitation therapy using LMC can apply patient-tailored therapy in virtual space and suggest an effective rehabilitation system that can confirm the therapeutic performance. Also, LMC can recognize ten fingers up to 0.01 mm and track motion at 200 frames per second, enabling precise operation. In addition, actual hand motion is recognized as the coordinate of the *Z*-axis representing the depth of field and depth of the super-wide angle of 150 degrees expressed in a 3D space. This makes it possible to accurately measure the motion of the same user as the actual one. LMC is a special device for hand movement recognition [34]. It consists of an infrared (IR) sensor for tracking 3D spatial coordinates. The IR sensor can track the information from hand joints so that the movement of the user can be tracked as it is. The recognition range of the LMC can obtain the position of each joint located on the hand (Figure 2).

**Figure 3:**
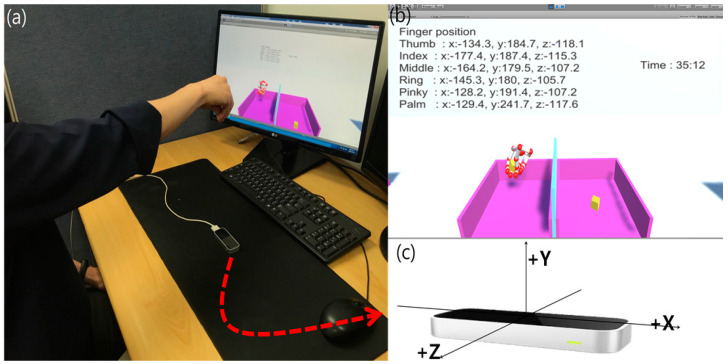
System BBT implemented in C # through Unity5.6.2p4 based on LMC. (**a**) Perform the developed system BBT in the same way as the existing conventional BBT. (**b**) When the system starts, the user can perform the system BBT efficiently through time and coordinate values. (**c**) 3-axis motion recognition of a real wrist joint based on LMC.

We used Unity5.6.2p4, a game engine, and integrated development environment, to visualize LMC in virtual space. Unity is a development tool primarily used in VR and AR content. It supports various platforms and specific functions basically. As a VR development platform, it is possible to create a 3D virtual space and display coordinates of the 3D virtual space tracked by LMC on the screen, including an additional engine that can interface with the LMC (Figure 3). The actual test had 150 blocks of wood. However, we performed two blocks to recognize the exact finger coordinates (Figure 3a). Two boxes, partitions, and wooden blocks were modeled in proportion to their actual size for use in virtual space by using BBT, the conventional rehabilitation therapy method. The 3D modeling work was performed using Auto Desk Inventor (Professional 2016, Autodesk, CA, USA). Next, 3D objects were implemented in 3D space using C-sharp (C #) through Unity.

When the system starts, a timer is displayed on the screen so that the user can check the time (Figure 3b). From the moment we first catch an object, 3D coordinates for the hand are stored so that the initial hand is recognized, and the position of the object is properly recognized (Figure 3c). In addition, when the block is moved to the left box, the number of the moving blocks is counted. When the block reaches the right box or falls outside, the user can return to the right box to perform a repetitive process. As with the actual conventional BBT, the subject is asked to move each block from one box to another for one minute, and the number of blocks moved is counted. After three training sessions to adapt to the system BBT, the user was tested and measured. The test was recorded in 60 s where one’s hand could move blocks to the opposite side. Also, when the number of blocks was counted, the hand must fall over the middle partition. When two blocks were pointed to at a time, they were counted as one. If the finger did not pass over the partition and the block was thrown away, the count was 0. After obtaining the wrist motion values of the user in the system BBT, the motion was analyzed with 3D graphing through MATLAB (The MathWorks, Natick, MA, USA). The test of this study was open-chain exercise (OCE). OCE was performed for the distal segment of the body segment (wrist joint). It is a suitable exercise for evaluating a defect in muscle performance. It is also an effective approach in early rehabilitation therapy [35].

### 2.4. Statistical Analysis

All statistical analyses were performed using SPSS version 21.0 (IBM, New York, NY, USA). It was assumed that there was no significant difference between conventional BBT and system BBT. The number of boxes moved in 1 min from the conventional BBT and system BBT were counted. In addition, Z-score standardization was performed to compare the data size of each group and make the data distribution similar to each other. Then, an independent *t*-test was performed (*p* < 0.05) to compare the means of the two methods.

## 3. Results

### 3.1. Evaluation System for Motion Tracking Analysis

Unlike existing rehabilitation therapy methods, we implemented BBT, an upper extremity rehabilitation therapy performed in virtual space. LMC can substitute expensive equipment because it is easy to implement individualized rehabilitation therapy. In addition, it can be repeatedly performed by the user alone, and the result of therapy can be seen. The system developed in this study can analyze the entire treatment process from start to finish of the movement by continually tracking the movement of the user’s hand while graphically displaying motion patterns when the user is performing system BBT. In addition, it can provide data to diagnose the patient with a small movement pattern during the test. This can be used as an objective indicator to identify the level of functional recovery by numerically representing the behavior of the patient undergoing rehabilitation therapy. In addition, unlike conventional upper limb rehabilitation system studies, it is a system that enables objective evaluation for diagnosis that is appropriate for the purpose of therapy. In addition, the user can perform rehabilitation therapy without space limitation in a comfortable environment because there is no need for expensive hardware equipment. Therefore, it is possible to implement home-rehabilitation programs that can be practiced at home by implementing the upper extremity rehabilitation therapy used in the virtual space. This can lead to a patient’s interest in a safe environment, an objective graph, and a clear goal awareness of the patient’s function recovery process.

**Figure 4:**
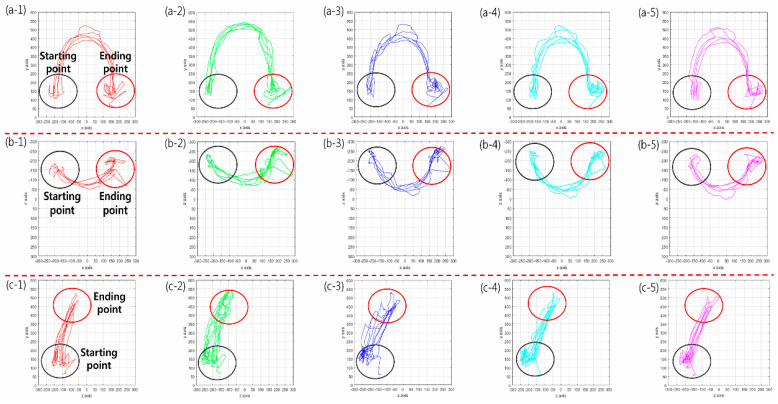
2D movement pattern using 3D coordinates (3-axis). (**a**)/(**1**–**5**) X–Y axis, (**b**)/(**1**–**5**) X–Z axis; (**c**)/(**1**–**5**) Z–Y axis. Red line: thumb (finger); green line: index; dark blue line: middle; sky blue line: ring; purple line: pinky. The start and endpoints can be displayed to determine the exact start and endpoints and the user can analyze wrist motion and depth using the 3D graph.

**Figure 5:**
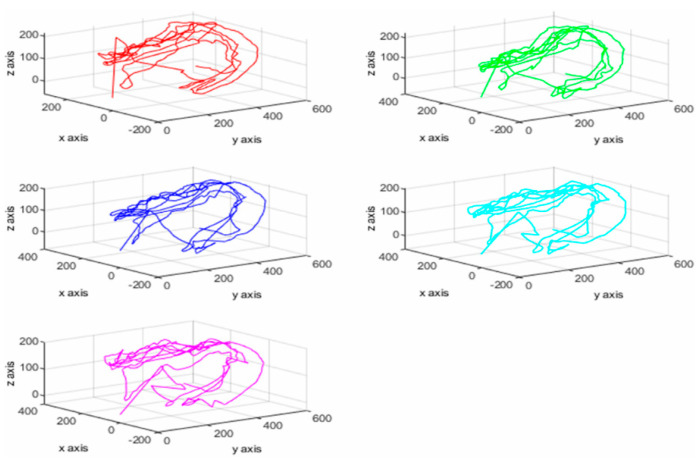
Tracking coordinates of the palm and fingers through the 3D graph. Red line: thumb (finger); green line: index; dark blue line: middle; sky blue line: ring; purple line: pinky. It is possible to analyze the upper extremity motor ability by tracking the movement of each finger of the user so that it can be presented as objective diagnostic evaluation data that conventional BBT cannot offer.

The result of the motion pattern can be obtained by 2D graphing with MATLAB using 3D coordinates of each finger obtained from the motion result of system BBT as shown in Figure 4. This shows the exact start and endpoints (Figure 4a–c). Also, the user can analyze the characteristics and depth of the wrist movement through the 3D graph. In particular, the thumb finger grips the object with the counter pressure (gravity) of the gathered thumb at the bending position of the finger at the wrist joint in the process of holding the box in the virtual space. At this time, mainly isometric movement is performed. The thumb is the most important part of gripping (opposition) to reinforce other fingers to help control the force for small movements. During the system BBT process, the user’s palms and five fingertips are tracked and a 3D graph using each axis can produce a variety of plan coordinates (Figure 5). After tracking the movement of the user and storing each coordinate value, the upper extremity motor ability can be judged. Thus, it is possible to present more objective diagnostic evaluation data than conventional BBT. The wrist joint is a two-axis (biaxial) joint capable of flexion/extension, abduction/adduction, and circumduction. The grip of the wrist is performed by the core muscles of the wrist joint. In particular, the strength of wrist extensor muscles and wrist contraction is proportional to the gripping force. At this time, wrist extensor muscles including extensor carpi radialis longus muscle against the flexor muscles are in a state where the origin-insertion portion is distanced by the length–tension force without being able to exert a sufficient force. This weakens the contraction force and results in a passive insufficiency state that prevents the wrist extensor muscles from stretching any further. The role of the wrist joint in gripping is to maintain the tension by the ligament of the wrist flexor/extensor muscles and to compensate to prevent muscle damage and rupture. The muscle spindle and Golgi tendon organs are responsible for length–tension change during muscle contraction/relaxation. A proprioceptor then transmits to the central nervous system and controls the muscle properly. Stroke patients can experience muscle rigidity due to these functional impairments. They are treated with a subjective judgment of movement patterns according to the therapist’s experience. Decision making based on the objective analysis of the movement pattern and behavior in rehabilitation therapy is an important factor that can show a difference in the quality of a patient’s life. The results of this study showed that when an abnormal pattern was generated by the 3D analysis in the user’s movement, the tracking result could be confirmed, and the motor ability could be judged.

**Figure 6:**
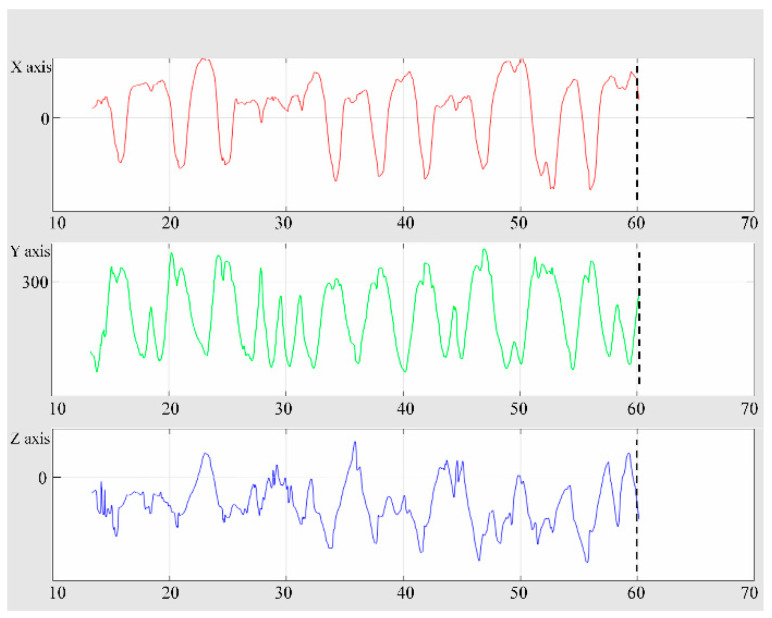
Ability to stretch the user’s hand and analyze the movement of each axis with time. The X axis according to time is width movement and Y axis is longitudinal movement. Also, Z axis means the depth of movement. The algorithm developed in this study enables stretch measurements (tremor) that could not be done in conventional BBT. It is also possible to analyze objective hand tremors, cerebral palsy, ataxia, and Parkinson’s disease.

Figure 6 shows the changes in each axis according to time. It can analyze the movement of the user according to the change in the distance that the user can reach. The X axis according to time is width movement and Y axis is longitudinal movement. Also, Z axis means the depth of movement.

**Figure 7:**
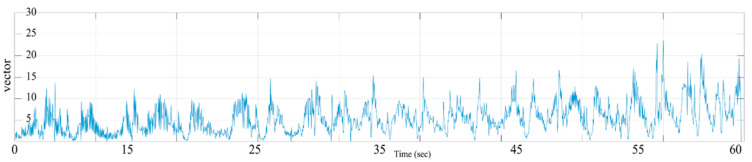
Performed tracking analysis of the user’s motion vector change as a repetitive activity. As time changes, it is possible to analyze the section where the vector changes. It is also possible to enhance the functional ability to move the box to the opposite box without making a mistake by stimulating the proprioceptor through repetitive activities of the user.

Stretch measurement is an efficient diagnostic method for ROM and muscle strength evaluation of patients with joint and muscle contraction. In addition, it can be an important indicator of objective hand tremor analysis of central nervous system diseases such as cerebral palsy, ataxia, and Parkinson’s disease. Figure 7 is a graph showing a motion vector change that moves a BBT block generated in the repetitive activity. Vector changes were set from the time the block was first held. The block is then moved to the opposite box. The repetitive section is ended as soon as it is worn to the floor. Starting from the moment, the next block is caught. It represents the motion vector value according to the number of repetitions. As the time changes, we can analyze intervals where the vector changes through repetitive activities. In addition, it means that the user has made a functional enhancement as repetitive activities can stimulate the user’s proprioception through the visual feedback of the section where the mistake is made so that the user can move the box to the opposite box without making a mistake. Clinically, repetitive activities (habituation) can promote new protein activity in a damaged brain [36]. Furthermore, repetitive activities can be based on the neuroplasticity theory that explains how changes in the excitability of neurons can cause the growth of new synapses in damaged axon dendrites. Thus, it can perform evidence-based rehabilitation therapy for the upper extremity.

### 3.2. Quantitative Comparison between System BBT and Conventional BBT

**Figure 8:**
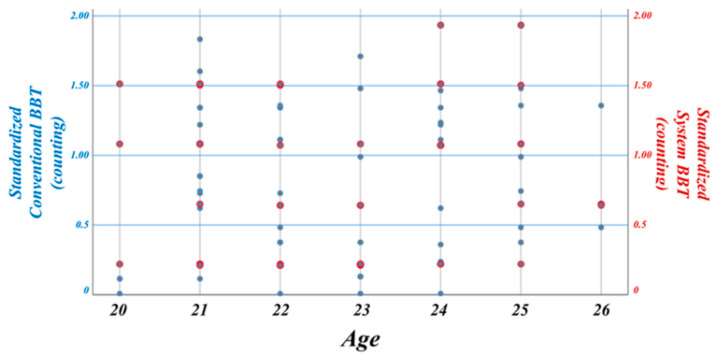
The standardization process in which data of each group are changed to the same size. Since it is difficult to compare each group’s data value, efficient comparison is possible after the Z-score standardization process.

**Figure 9:**
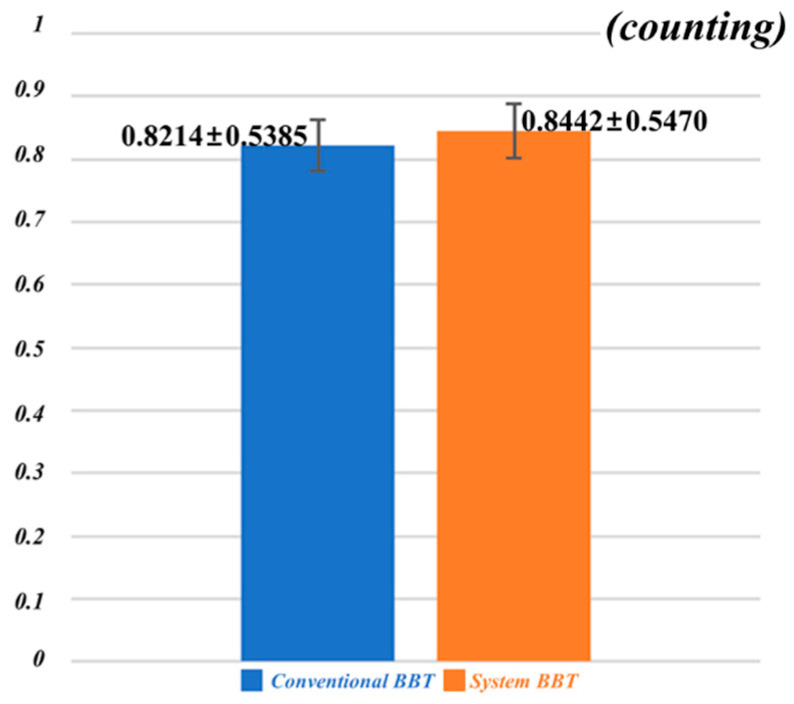
After standardization, independent *t*-test analysis is performed for mean values of the conventional BBT and system BBT. Data are expressed as mean ± standard deviation (n = 48). Conventional BBT (0.8214 ± 0.5385) and system BBT (0.8442 ± 0.5470) were not statistically significant. Results of this study can be used to analyze a possible home-rehabilitation program and motion pattern recognition at home to show the possibility of objective diagnostic evaluation data.

We performed a Z-score standardization process to make the distribution of data similar in order to show how the data of each group were different in terms of differences in the mean standard. Data of the two groups can then be viewed on the same basis (Figure 8). The Z-score standardization Equation (1) is as follows:

(1) $$Z=\frac{\left(X-m\right)}{\sigma }$$

where *X* is a numerical value, *m* is an average, and *σ* is a standard deviation. The result of the standardization process of conventional BBT and system BBT implemented in AR is shown in Figure 9. Data are expressed as mean ± standard deviation (n = 48). The difference between the mean value of the conventional BBT (0.8214 ± 0.5385) and the mean value of the system BBT (0.8442 ± 0.5470) was not statistically significant. This result indicates that system BBT is effective in rehabilitation therapy for the upper extremity. In addition, existing therapies are limited by the high cost of rehabilitation therapy, the visual judgment of the therapist, limited space, and so on. The results of this study can be used to analyze possible home-rehabilitation programs and motion pattern recognition at home to show the possibility of objective diagnostic evaluation data.

## 4. Discussion

We enrolled 48 healthy participants (23 males and 25 females) in their twenties (22.71 ± 1.65 years) in order to objectify movement tracking and diagnosis of patients after tracking the upper extremity rehabilitation therapy model in virtual space using LMC. Our results demonstrate that the system BBT is a quantitative diagnostic evaluation method by providing an objective judgment index using a coordinate change of each finger and palm of the hand through user motion tracking in 3D virtual space. In the rehabilitation field until now, the patient’s movement has been monitored using physical devices. Subjective analysis is then performed by the therapist for diagnosis and evaluation. Therefore, it is difficult to analyze the effect of individual therapy due to the different environments of each patient [37]. In addition, a variety of assistive devices and training used for upper extremity rehabilitation therapy were mainly used in rehabilitation hospitals equipped with actual therapy equipment. The previous evaluation method was confined to a clinical aspect with the limitations of objectivity and quantitative factors for diagnostic evaluation. The existing subjective assessment provides difficulties for the therapist to make an accurate diagnosis. In addition, there is a limit to the objective evaluation of the patient. Moreover, the degree of loss of motor function is an important indicator for the prognosis of stroke patients.

However, it is difficult to objectively evaluate the complexity of muscle tension, and synergy movement and associated reaction. Therefore, rehabilitation therapy should be made objective and accurate with a state change of the current therapy process. During activities of daily living (ADL), the hand function of the upper extremity region is the most important part [6,7]. Behavioral analysis requires objective and consistent movement tracking [38]. Therefore, hand therapy requires sophistication and focuses on starting with small tools. The goal should be to expand through various processes such as independent activities in order to obtain delicate movements that can help with ADL. A natural hand gesture allows one to perform basic movement necessary for ADL through repetitive training. Further work can be applied in clinical practice by linking a haptic element, which enables the user to feel the touch of the box when operating in the AR, with LMC [39]. Clinically, repetitive activities can reduce the release of excitatory neurotransmitters including glutamate and promote brain activation. Through repetition of specific stimuli, the synthesis and activation of new proteins can induce excitatory changes in neurons and promote the growth of new synapses in dendrites [40]. In addition, brain activation can be achieved by movement through constraint-induced movement therapy of the affected upper extremity and an evidence-based approach as a repetitive activity of holding hands. Therefore, it is possible to provide an objective performance index by accurate motion analysis of a patient through a subsequent clinical study. Compared with the subjective evaluation of rehabilitation medicine specialists and therapists for existing therapy outcomes, this system quantifies results such as tracking speed of motion trajectory and position error out of the trajectory so that the possibility of objective and accurate judgment can be presented. The final goal of the therapist functional recovery through rehabilitation therapy. The results of this study can be used as basic data for the diagnostic evaluation of evidence-based degenerative neurology.

## 5. Conclusions

LMC-based rehabilitation therapy has a limit in that it only performs analysis based on the visual judgment of the therapist using only existing contents. However, the system BBT developed in this study showed similar results to the actual conventional BBT. The objective graph of the user’s movement pattern showed that the evaluator could quantitatively analyze it. Therefore, system BBT can be applied to nervous system patients in the clinic so that the therapist can recognize any abnormal pattern based on objective data. The results of this study can also be used as basic data for evidence-based diagnostic evaluation. The future work can be applied to medical research that can be evaluated by classifying the degree of recovery according to the type of upper extremity disease by recording the tracked wrist joint of the developed system BBT.
